# Professionalizing Healthcare Management: A Descriptive Case Study

**DOI:** 10.15171/ijhpm.2017.40

**Published:** 2017-04-09

**Authors:** Erika L. Linnander, Jeannie M. Mantopoulos, Nikole Allen, Ingrid M. Nembhard, Elizabeth H. Bradley

**Affiliations:** ^1^ Yale School of Public Health, Yale University, New Haven, CT, USA.; ^2^ Yale Global Health Leadership Institute, Yale University, New Haven, CT, USA.

**Keywords:** Healthcare Management, Health Policy, Quality Improvement, Low- and Middle-Income Countries (LMICs)

## Abstract

Despite international recognition of the importance of healthcare management in the development of high-performing systems, the path by which countries may develop and sustain a professional healthcare management workforce has not been articulated. Accordingly, we sought to identify a set of common themes in the establishment of a professional workforce of healthcare managers in low- and middle-income country (LMIC) settings using a descriptive case study approach. We draw on a historical analysis of the development of this profession in the United States and Ethiopia to identify five common themes in the professionalization of healthcare management: (1) a country context in which healthcare management is demanded; (2) a national framework that elevates a professional management role; (3) standards for healthcare management, and a monitoring function to promote adherence to standards; (4) a graduatelevel educational path to ensure a pipeline of well-prepared healthcare managers; and (5) professional associations to sustain and advance the field. These five components can to inform the creation of a long-term national strategy for the development of a professional cadre of heathcare managers in LMIC settings.

## Background

Effective healthcare management is essential for the creation of a high performing healthcare delivery system.^1-3^ A number of studies have demonstrated the link between management capacity and health systems performance,^4-11^ and the lack of managerial capacity at all levels has been cited as a key constraint in the achievement of the Millennium Development Goals and other global health targets.^12^ Healthcare management – defined as the process of achieving healthcare objectives through human, financial, and technical resources^13^ – includes strategic and operational management activities such as supply chain management, human resources management, performance management and improvement, financial management, and governance, without which resources cannot be effectively deployed to maximize health outcomes.^14^

Healthcare management is particularly critical in the public sector of low- and middle-income countries (LMICs) where resources are inadequate and efficiency in their deployment is essential to meet the national and global targets for health outcomes. In many LMIC settings, physicians are called upon to fill healthcare management roles because of their level of education, respected status in society, and clinical/technical expertise related to the services being offered.^15,16^ However, physicians typically lack previous management-related training, mentorship, and professional development^15^ that would prepare them for these roles. Clinicians in managerial roles are often asked to simultaneously continue their clinical practice, a dual role associated with low job satisfaction, burnout, and attrition from the workforce.^17,18^

Despite international recognition of the importance of healthcare management in the development of high-performing systems,^1-3^ and the positive association between managerial training programs and healthcare quality,^19-22^ the path by which countries may develop and sustain a professional healthcare management workforce has not been articulated. Accordingly, we sought to identify a set of common themes for establishing and sustaining a professional workforce of healthcare managers in LMIC settings using a descriptive case study approach. This paper can catalyze reflection on broader efforts to build health care management capacity, and guide LMICs as they consider alternative strategies for health management workforce development as a critical component of health systems strengthening.

## Setting

Over the past 10 years, Ethiopia has achieved significant progress toward global health targets, and is credited with ambitious investment in both hospital quality and access to primary care.^23-25^ To promote these improvements, the Ministry of Health prioritized the development of management capacity, with initial emphasis on hospital management.^26^ This 10-year arc of national reforms provide an ideal context in which to study the emergence of a professional cadre of health care managers in an LMIC setting.

## Methods

Using a descriptive case study approach,^27,28^ we sought to compare the path of the development of the profession of healthcare management in Ethiopia with experiences in the professionalization of healthcare management in the United States. We selected these two countries as paradigmatic cases^29^ through which to identify convergent and divergent experiences between country settings, resulting in a set of themes for future testing.

To understand the history of healthcare management in the United States, we conducted a semi-structured review of the peer-reviewed and grey literature, with emphasis on historical descriptions from healthcare management textbooks and published historical syntheses provided by relevant professional associations. To describe the more recent history of healthcare management in Ethiopia, we drew on a decade of our experience implementing and evaluating efforts to build management capacity in Ethiopia.^5,7,30-32^ We supplemented this experience with study of the peer-reviewed literature, starting with country-specific searches for “health management” and “healthcare management,” reviewing abstracts to identify relevant resources, and following up on references as appropriate.

## Common Themes in Professionalization

Many prominent development organizations, including the World Health Organization (WHO), the World Bank, and numerous others, have attempted to address gaps in healthcare management capacity through in-service training and mentoring programs.^15,19^ While short-courses and applied training programs can be a helpful stopgap for the healthcare professionals who find themselves in managerial roles, we envision a broader, more strategic set of common themes in the transition toward a comprehensively prepared, professional workforce of health managers (Box 1).

**Box 1. Common Themes in the Professionalization of Healthcare Management**

1. A country context in which healthcare management is demanded
2. A national framework that elevates a professional management role
3. Standards for healthcare management, and a monitoring function to promote adherence to standards
4. A graduate-level educational path to ensure a pipeline of well-prepared healthcare managers
5. Professional associations to sustain and advance the field

### Theme 1: A Country Context in Which Management Expertise Is Demanded

The first common theme in the professionalization of healthcare management in the United States and Ethiopia was a shift in country context toward *increased demand for the expertise of a cadre of health managers*. In the United States, calls for the professionalization of healthcare management began in the early 1900s as medical discoveries (eg, the advent of antisepsis and anesthesia, the development of modern surgery, and the discovery of antibiotics) attracted patients to seek medical care in hospitals as opposed to being treated at home.^33,34^ During this time, the hospital industry in the United States grew from 4 hospitals and 780 beds per million people in 1875 to about 60 hospitals and 7400 beds per million people in 1925.^33,35^

The severe economic constraints of the Great Depression in the late 1920s and early 1930s accentuated the need for healthcare administrators who could apply business acumen in the large and growing hospital sector. In 1932, The Committee on the Costs of Medical Care highlighted this evolving need for more sophisticated management of medical care^33^:

“*Hospitals and clinics are not only medical institutions, they are also social and business enterprises, sometimes very large ones. It is important, therefore, that they be directed by administrators who are trained for their responsibilities and can understand and integrate the various professional, economic, and social factors involved.*”

Academics took up the challenge as Michael Davis from the University of Chicago published *Hospital Administration, A Career: The Need for Trained Executives for a Billion Dollar Business, and How They May Be Trained* in 1929.^33^ The hospital industry trade journals followed suit, underscoring the increasing pressure to manage dwindling resources. As Malcom Meacher wrote in 1932 in the Bulletin of the American Hospital Association (AHA):

*“Stock crashes, bank failures, frozen assets, depreciated earnings, diminished benefactions, together with lessened payment by patients and decreasing departmental earnings and all that comes in the wake of these, have depleted the hospital treasury. Hospital trustees and executives are staggering under the valiant attempt to maintain adequate standards in order to insure safe and efficient care of their patients.”* ^36^

In Ethiopia, the demand for the expertise in healthcare management was driven by several factors. First, having met basic standards for *access* to primary care, the Ministry of Health shifted its focus to promoting the *quality* of medical care, particularly as a growing middle class demanded better healthcare services. Increasing demand for management expertise has also resulted from Ethiopia’s efforts to decentralize governance and control of finances to the hospital and district health office level improve healthcare system responsiveness and efficiency.^37,38^ Additionally, the increasing complexity of the health sector (eg, the introduction of third party financing, quality monitoring, and growing regulatory efforts) required more nuanced managerial approaches to successfully navigate. Taken together, these contextual factors generated demand for managerial competencies and set the stage for a movement toward professionalization—a movement supported by investment in the next four thematic areas.

### Theme 2: A National Framework That Elevates a Professional Management Role

The second common theme in the professionalization of healthcare management in the United States and Ethiopia was a *national framework or set of supporting policies that elevated the professional management role* to attract, empower, and reward management expertise. As in many LMIC settings today, most hospitals in the United States in the 1920s and 1930s were led by clinicians who acquired administrative responsibilities with no formal training or experience in administration.^33^ This later evolved into the esteemed Chief Executive Officer (CEO) position, a highly selective role with authority to lead and affect change in the organization.

An exemplar of this type of reform comes from Ethiopia, where a 10-year investment in hospital management was grounded in reform of civil service regulations to achieve the following: (1) creation of a full-time CEO role with clear and comprehensive job responsibilities, as well as selection and performance review criteria,^26,30,39^ (2) establishment of governing boards to manage CEO performance and hold the position accountable to both the government and the community,^32^ and (3) creation of locally-controlled revenue streams to reward good management and entrepreneurialism (eg, the endorsement of private wings in Ethiopia’s public hospitals and policies to allow for local retention of revenue for future health system investment).^40-43^ This CEO role, shown to be fulfilled successfully by physicians or non-physicians,^44^ presumes full-time dedication to management and leadership functions, involving both internal problem solving and strategic management of external community environments.^26,30,32,36,39-44^

### Theme 3: Standards for Healthcare Management, and a Monitoring Function to Promote Adherence to Standards

As professions emerge, a *set of technical, ethical, and/or performance standards* are used to define expectations for the profession.^45^ In the United States, the earliest performance standards for healthcare management were codified at the organizational level (hospital accreditation), rather than the individual level (licensure). Professional healthcare managers were expected to create and maintain hospital management systems in compliance with accreditation standards.

Hospital accreditation was first established in the United States by the American College of Surgeons (ACS) in 1917.^46^ The ACS’s “Minimum Standard for Hospitals” began as a single page of requirements, and evolved to become today’s Joint Commission, an independent accrediting body which authorizes over 21 000 healthcare organizations and programs in the United States.^47^ Since the early 1990s, accreditation has been adopted in a number of LMICs as a strategy to improve basic health service quality.^48^ Some of these accreditation programs have been launched as part of larger “pay for performance” financing reforms^49-54^; others have emerged as government-led efforts to directly quantify and improve management capacity in meaningful ways.

In Ethiopia, the first set of hospital management standards (referred to as “the Blueprint”) were derived directly from government hospitals through the collaborative experiences of foreign healthcare management mentors and their local counterparts as part of the “Ethiopian Hospital Management Initiative,” a multi-year collaboration between the Federal Ministry of Health, the Clinton Health Access Initiative, and Yale University to improve hospital quality.^30^ These management standards were subsequently endorsed by the Ministry of Health as the “Ethiopian Hospital Reform Implementation Guidelines” (EHRIG). Adherence to the EHRIG standards is now incorporated into Ethiopia’s hospital performance monitoring system as the first of 36 key performance indicators on which hospitals are evaluated by Ministry of Health officials.^55-57^

### Theme 4: A Graduate-Level Educational Path to Sustain a Pipeline of Well-Prepared Healthcare Managers

The fourth common theme in the United States and Ethiopia was the establishment of a *well-respected, graduate-level educational path to attract, equip, and sustain a high-level professional cadre*. The educational path should provide both didactic and practical preparation. Such a combination of classroom studies and fieldwork was used in the earliest healthcare administration programs in the United States, and continues today. The first bachelors-level program was established at Marquette University in 1926, but the professional status of the field was elevated when, in 1934, the University of Chicago developed the first graduate program in hospital administration based on the model of one year of coursework followed by one year of practical experience called an administrative residency. Other universities replicated this model, and the number of graduate programs in healthcare administration grew from 9 programs in the 1940s to 18 programs by the 1950s and 33 programs by the 1960s.^58^ In 1968, the Accrediting Commission on Graduate Education for Hospital Administration, known today as the Commission on Accreditation of Healthcare Management Education, was incorporated as the accrediting agency for graduate programs in health administration. Today, the United States has 88 accredited graduate programs in healthcare management and administration.^59^

In many LMICs, graduate-level educational programs in healthcare management are few in number.^19-21,60^ As a cornerstone of their hospital reform efforts, and with initial focus on the newly-created CEOs described above, Ethiopia established its first Masters in Healthcare Administration (MHA) at Jimma University in 2009. By 2016, MHA programs were offered by five public universities across the country.^7,22,39^ These curricula combined didactic education and mentored independent fieldwork, and engaged well-respected national and international academic institutions. As a signal of political commitment to the profession and the individual participants, the Ministry of Health covered the costs of participation in early student cohorts.

### Theme 5: Professional Associations to Sustain and Advance the Field

Professional associations provide both the *networking and career development opportunities* required for a new cadre of professional health managers to continue to elevate, advocate for, and sustain their roles within countries. In the United States, the first professional association in hospital administration dates back to 1899, when hospital superintendents, predecessors to the CEO role, came together to understand and navigate their increasingly complex US healthcare landscape. Even before the creation of the first graduate program in hospital administration in the United States, a group of practicing administrators founded the American College of Hospital Administrators in 1933 (now the American College of Healthcare Executives [ACHE]), with an emphasis on non-clinical administrators.^58^ Today, ACHE boasts almost 50 000 members across 79 chapters, offers board certification for healthcare executives, and convenes members for continuing education, networking, and mentoring through both an annual Congress and regional chapter events, all in service to the holistic development of the profession.^61,62^

In contrast, investments in healthcare management in the Ethiopian context have been driven primarily by the Ministry of Health and development partners. MHA program alumni have come together to form the country’s first Ethiopian Society of Healthcare Administrators to advocate for the profession, influence supporting policy, and promote continuing professional development opportunities. Professional associations like this one may help to promote the continued growth and evolution of the newly-established profession as it gains a foothold, but this potential impact is yet to be demonstrated.

## Discussion

This paper outlines five components that can inform the creation of a long-term national strategy for the development of a professional cadre of healthcare managers in LMIC settings. The five common themes – a demand for management expertise, elevation of the management role, standards for healthcare management systems, a graduate-level educational path, and professional associations – are synergistic. This type of holistic strategy, currently exemplified in Ethiopia, is unlikely to emerge without exceptional country leadership to align health policy reform, development partner investment, and university engagement. Commitment at all levels is critical, as large-scale shifts in professional trajectories take time, and investments in healthcare management capacity challenge the status quo on multiple levels^1^ as empowered managers begin to advocate more proactively with government officials and clinical professional groups.

At the same time, it is important to consider the potential for unintended effects of professionalization. A more empowered cadre of healthcare executives has the potential to overpower the community voice in healthcare reform.^63,64^ That said, community and client engagement is at the heart of effective management practices, and approaches to amplify the perspectives of community members (eg, the use of community scorecards and creation of community member seats on governing boards) are commonly promoted in management education and professional standards. Professionalization may also result in fragmentation in the authority for management and clinical objectives.^65^ Accordingly, management education and career development programs must equip healthcare managers to work effectively across boundaries between management and clinical professionals. In high-income countries, healthcare organizations include clinicians and non-clinicians in both senior management and governing board roles, and quality improvement projects and approaches commonly bridge professional and organizational boundaries to address complex challenges in pursuit of more effective healthcare. Ultimately, professionalization requires devolution of managerial authority, a power that could be corrupted. Our hope is that by establishing a national-level strategy, alignment can be found between regulatory, financing, education, and service delivery systems to create checks and balances across functions.

Our findings should be interpreted in light of the limitations of the study design. First, we highlighted a set of common themes rather than a causal explanation of how healthcare management becomes professionalized. Future prospective studies may be useful to assessing causal inferences. Nevertheless, the patterns identified provide useful insights about the professionalizing of healthcare management, and our findings are consistent with sociological study of professionalization in other technical fields, which emphasizes specialized training and expertise, authority and autonomy, regulation, and creation of group identity.^66-68^ Second, our results are based on historical analysis and implementation experiences in two country settings, and results may differ in other country contexts. We anticipate that the specific design and timing of various components of professionalization must be tailored to the unique context of each country.^23^ For example, in the United States, hospital standards generated by the ACS, as well as the establishment of the AHA and ACHE were early drivers of professionalization for healthcare managers. In other country contexts in which professional lobbies are not well developed or even discouraged, reform will likely start through centrally-driven initiatives. Additional implementation science research is required to understand the extent to which these themes are consistent with experiences in other LMIC country settings, and to evaluate the potentially synergistic impact of investment in some or all of these thematic areas.^69^

The five interrelated themes presented here, identified through historical analysis and implementation experience across two very different country settings, may serve as guideposts as LMICs seek to move from discrete investments in management capacity toward a more strategic, sustainable plan for the development of a professional healthcare management workforce. A professional cadre of healthcare managers, able to effectively balance broader regulatory, financial, and service delivery reforms, is a solid foundation on which to build better health systems and, ultimately, improved health outcomes.

## Ethical issues

Not applicable.

## Competing interests

Authors declare that they have no competing interests.

## Authors’ contributions

ELL and EHB conceived of the manuscript; all authors made substantive contributions to the perspective and reviewed and approved the final manuscript.

## Authors’ affiliations

^1^Yale School of Public Health, Yale University, New Haven, CT, USA. ^2^Yale Global Health Leadership Institute, Yale University, New Haven, CT, USA.
